# Gyroscopic Radiosurgery as a Non-invasive Alternative Treatment for Maxillary Sinus Squamous Cell Carcinoma: A Case Report and Literature Review

**DOI:** 10.7759/cureus.86153

**Published:** 2025-06-16

**Authors:** Pablo Mazon, Alec Zheng, Xiaodong Wu, Aizik Wolf

**Affiliations:** 1 Miami Neuroscience Center, Larkin Community Hospital, Miami, USA; 2 Neurological Surgery, Hospital Universitario Insular de Gran Canaria, Las Palmas, ESP; 3 Department of Neuroscience and Behavioral Biology, Emory University, Atlanta, USA; 4 Medical Physics, Executive Medical Physics Associates, Miami, USA

**Keywords:** head and neck squamous cell carcinoma (hnscc), maxillary sinus carcinoma, sinonasal malignancy, stereotactic radiosurgery srs, zap-x radiosurgical system

## Abstract

Squamous cell carcinoma (SCC) of the maxillary sinus is a rare malignancy that often presents at an advanced stage due to its nonspecific symptoms and anatomical complexity. Surgical resection remains the primary treatment, frequently combined with radiotherapy and/or chemotherapy. However, in selected patients, non-surgical alternatives may be necessary due to comorbidities or personal preference. We report the case of a 46-year-old male with a history of pharmacologically managed hypertension who presented with progressive left ocular discomfort, erythema, and epiphora. Imaging revealed a mass occupying the left maxillary sinus with regional extension. Histopathological analysis confirmed SCC. Although extensive surgical resection was recommended, the patient declined surgery and chemotherapy due to concerns about functional and cosmetic outcomes. As an alternative, he underwent stereotactic radiosurgery using the ZAP-X system. The treatment delivered a total of 27 Gy in three fractions with conformal dose distribution and strict organ-at-risk sparing. Follow-up over 12 months demonstrated sustained clinical improvement and radiological evidence of complete response, without evidence of local recurrence or distant metastasis. While surgery followed by adjuvant therapy is the gold standard for maxillary sinus SCC, this case supports the potential role of stereotactic radiosurgery as a viable, organ-preserving alternative in early-stage disease when surgery is not feasible or preferred. A review of the literature indicates limited data on non-surgical management for early-stage SCC, particularly using stereotactic radiosurgery systems like ZAP-X. This case is the first reported instance of early-stage (T2N0M0) maxillary sinus SCC successfully managed with stereotactic radiosurgery alone, demonstrating favorable clinical and imaging outcomes after one year. This treatment strategy may offer a promising alternative in well-selected cases, warranting further research into its broader applicability.

## Introduction

Malignancies of the nasal cavity and paranasal sinuses are rare entities, with an estimated annual incidence of one case per 100,000 individuals [1-3]. Tumors arising in the paranasal sinuses account for only 0.2-0.8% of all malignancies, with the maxillary sinus being the most frequent site (60% of cases) [1,4,5]. Squamous cell carcinoma (SCC) of the maxillary sinus represents approximately 1% of all cancers and remains the most common histopathological subtype, constituting up to 80% of diagnosed cases [6].

The clinical presentation of these tumors is often nonspecific, leading to misdiagnosis as allergic, inflammatory, or infectious conditions. This diagnostic ambiguity contributes to delayed detection and advanced-stage presentation [1,6]. Common symptoms include bloody nasal discharge, non-healing mucosal lesions, facial swelling, trismus, unilateral nasal obstruction, and ocular discomfort [6].

Treatment options vary according to the characteristics of the tumor, with surgery being the cornerstone of treatment for resectable maxillary sinus SCC. Early-stage tumors may be managed with endoscopic resection, while advanced cases often require open approaches such as maxillectomy or wide craniofacial resection [6]. Adjuvant radiotherapy (RT) is standard for tumors with positive margins or high-risk features to reduce locoregional recurrence. For advanced-stage disease, combined chemoradiotherapy demonstrates superior survival outcomes compared to monotherapy [6,7].

Herein, we present a case of a male patient with maxillary sinus SCC who declined surgery and chemotherapy, receiving gyroscopic radiosurgery using the ZAP-X system, with a one-year follow-up period demonstrating favorable clinical outcomes.

## Case presentation

A 46-year-old male with a medical history of hypertension managed pharmacologically presented with a one-year history of progressive left ocular discomfort, erythema, epiphora, and periorbital pain. Initial clinical evaluation suggested a left lacrimal gland obstruction, prompting referral for surgical intervention. Postoperative biopsy revealed histopathological findings consistent with SCC, HPV positive, PD-L1 positive, and the patient was referred for further oncological workup.

**Figure 1:**
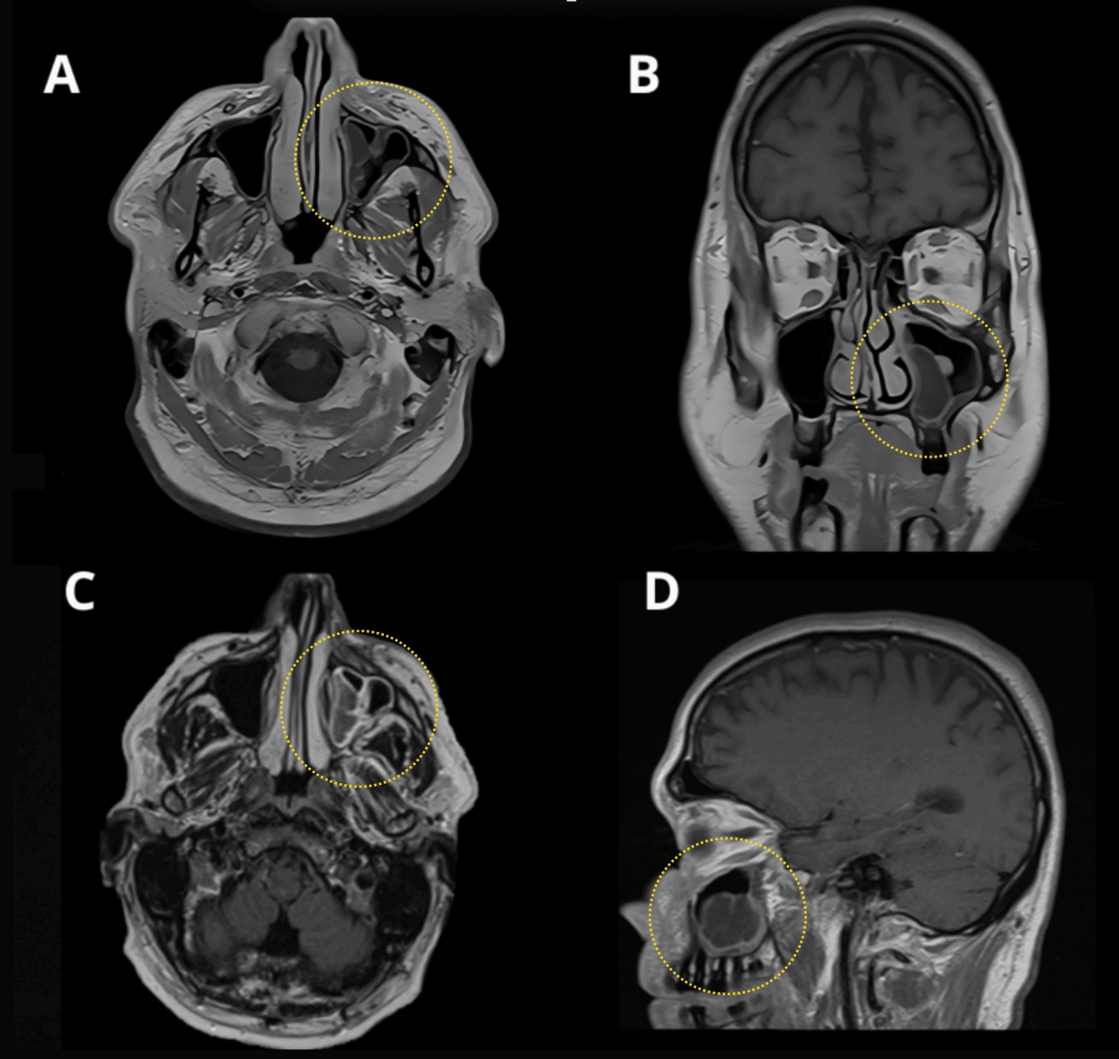
Pre-treatment head MRI with and without contrast. A. Axial T1-weighted image without contrast showing mucosal thickening of the left maxillary sinus. B. Coronal view demonstrating partial obliteration of the medial zone of the left maxillary sinus with mucosal thickening and altered signal intensity. C. Axial T1-weighted image with contrast revealing peripheral enhancement of the lesion within the left maxillary sinus. D. Sagittal view without contrast showing altered signal intensity of the mucosa in the left maxillary sinus.

Post-diagnosis, contrast-enhanced MRI of the head demonstrated a heterogeneously enhancing mass occupying nearly the entire left maxillary sinus, with extension to the left osteomeatal complex, inferior orbital wall, and complete opacification of the left maxillary sinus (T2) (Figure 1).

Subsequent staging PET/CT identified a hypermetabolic lesion centered at the left maxillary sinus, involving the left medial canthus and left lacrimal sac/duct. Additionally, scattered FDG-avid reactive cervical lymph nodes were noted.

The diagnosis of maxillary SCC T2N0M0 (stage II) was made, and the patient underwent comprehensive evaluation. Surgical intervention was recommended, involving left orbital exenteration, radical maxillectomy, and bilateral neck dissection. However, the patient declined extensive resection due to concerns about functional and cosmetic morbidity, opting instead to pursue non-surgical and non-systemic alternatives. For this reason, radiosurgery was offered and subsequently chosen despite having been previously informed of the potential risk of recurrence that may result from receiving this treatment alone.

**Figure 2:**
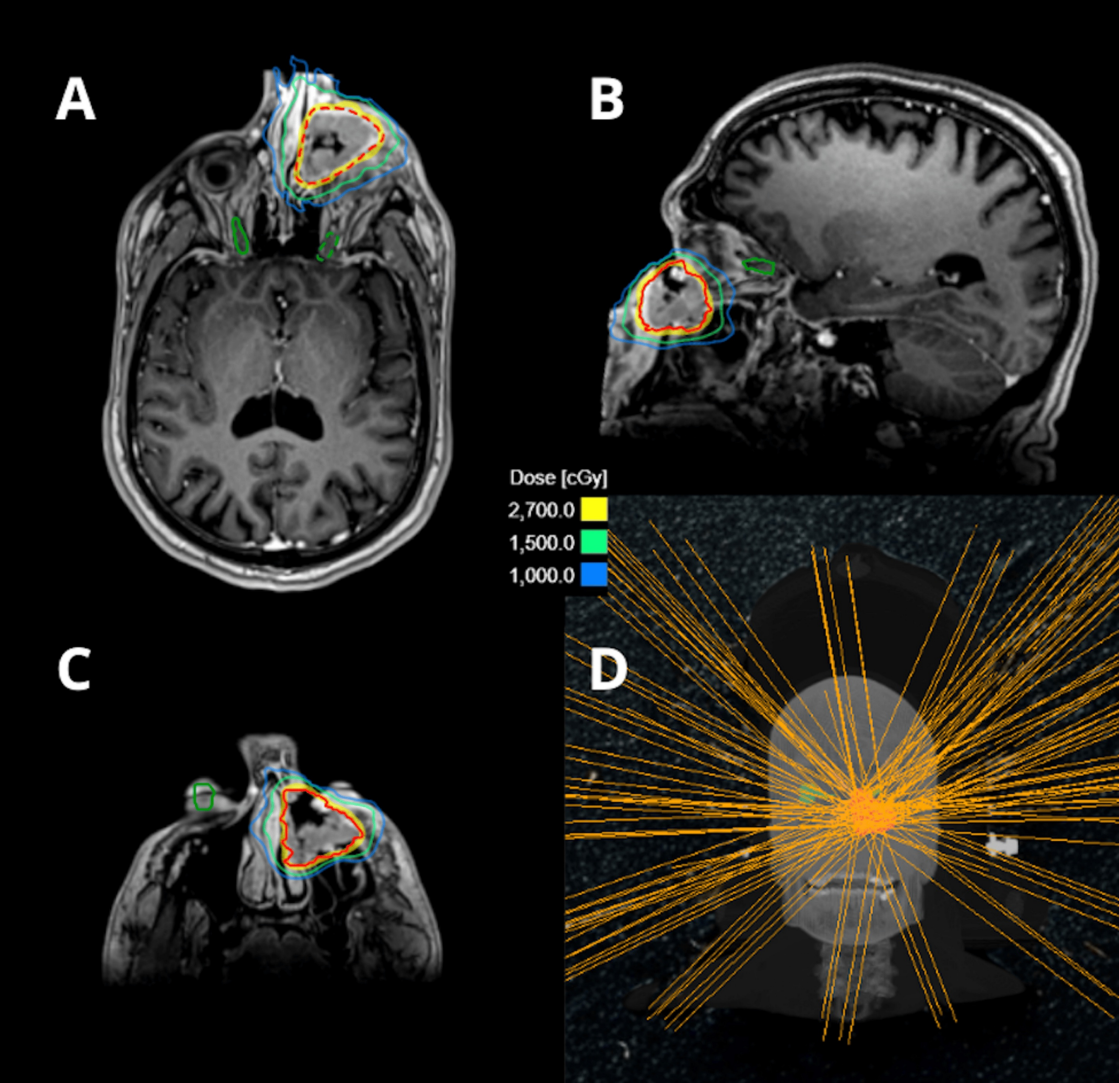
ZAP-X treatment delivering 9Gy x3 to the tumor volume using contrast-enhanced T1-weighted brain MRI. A, B, C. Axial and sagittal views illustrating the dose coverage of the target volume while preserving adjacent critical structures. View delineating the lesion to be treated, marked with a red dotted line. D. Beam angle distribution showing the 139 trajectories utilized by the system to irradiate the tumor.

The patient was scheduled for three sessions of stereotactic radiosurgery using the ZAP-X system. The target volumes and critical structures were specified and delineated in the ZAP-X treatment planning system, incorporating MRI and CT fusion for precise target definition. The treatment plan was designed to deliver 9 Gy × 3 to the 55% isodose line covering the tumor, which measured 18.5 cc, using 12 isocenters and 139 beams with 12.5 mm, 15 mm, 20 mm, and 25 mm cones, totaling 17,414.13 MUs. Doses to critical structures were minimized, particularly to the optic pathway, which received no more than 5.62 Gy, and to the right lens, which received no more than 1.52 Gy (Figure 2). This dose was selected based on the consideration that 9 Gy × 3 fractions is biologically equivalent to a single 18 Gy fraction using α/β = 10 Gy [8], which is regarded as an ablative radiosurgical dose level, while also aiming to minimize toxicity to the sensitive surrounding tissues.

The patient underwent a one-year follow-up after treatment, with clinical and imaging evaluations every three months. During follow-up, the patient reported significant improvement in left ocular pain and epiphora. Physical examination revealed resolution of left conjunctival erythema and stable cervical lymphadenopathy. Three months after treatment, a restaging PET/CT was performed, showing significantly decreased metabolic activity and size of the maxillary SCC, along with decreased metabolic activity in previously FDG-avid cervical lymph nodes. There was no evidence of distant metastatic disease. A brain MRI was also performed, which showed no evidence of intracranial metastatic disease and only partial opacification of the left petrous and mastoid air cells.

**Figure 3:**
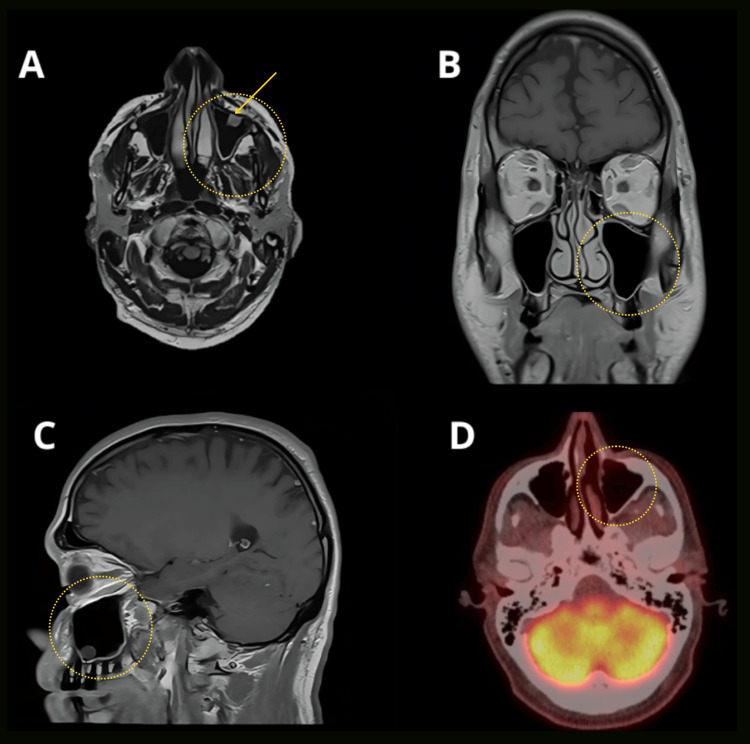
One-year post-treatment brain MRI with and without contrast, and PET/CT imaging. A. Axial T1-weighted image with contrast showing no mucosal thickening or residual lesion; a mucosal retention cyst is indicated by the arrow. B and C. T1-weighted images without contrast demonstrating different views of the left maxillary sinus without mucosal thickening or evidence of residual pathology. D. PET/CT scan revealing physiologic distribution of FDG in the head, with no abnormal uptake at the left maxillary sinus.

One year after treatment, another PET/CT was performed, demonstrating physiologic distribution of FDG activity in the brain, no suspicious increased FDG uptake in the region of the left lacrimal sac, and complete resolution of FDG avidity in previously noted reactive lymph nodes. Head-brain MRI showed stable mild subcutaneous soft tissue thickening in the medial premaxillary soft tissue on the left side, with no associated enhancement - findings likely representing post-treatment changes. A 1.2 cm mucosal retention cyst was observed in the left maxillary sinus, and the mastoid air cells appeared clear (Figure 3).

## Discussion

The treatment of SCC of the maxillary sinus is complex due to anatomical challenges and the advanced stage at which these cancers are often diagnosed. The most effective treatment options typically involve a combination of surgery, radiotherapy, and chemotherapy, with the choice of modality influenced by the stage of the disease and the patient’s overall health.

The literature supports surgery as the first-line treatment for these types of lesions, often accompanied by adjuvant therapy. Zhang et al. conducted a retrospective study of 60 cases, comparing three different treatment modalities. The five-year survival rates were 51.7% for patients treated with radiotherapy plus surgery, 33.3% for those receiving concurrent chemoradiotherapy with adjuvant surgery, and 18.2% for those treated with radiotherapy alone. Tumor staging of the cases was not specified throughout the study [9].

Lee et al. in 2023 conducted a review of 1788 patients, 71.2% of whom were classified as stage IVA, and concluded that neoadjuvant treatment followed by surgery, as well as adjuvant treatment following surgery, were associated with the highest adjusted five-year survival rates for stage IVA-B tumors. Surgery alone also demonstrated favorable survival outcomes, though not as high as the combination treatments. Radiation alone showed the highest hazard ratio and the lowest adjusted five-year survival rate, indicating that it was the least effective treatment option in terms of survival [7].

Jaimati et al. reported in 2014 on a retrospective study of 30 patients, 53% of whom were at advanced stages (IV) and received surgery followed by postoperative radiotherapy. The overall five-year survival rate was 65.7% for patients who underwent radical surgery followed by adjuvant radiotherapy, with a recurrence rate of 23.3%. This suggests that while the treatment was effective for a majority of patients, a significant portion experienced disease recurrence [10].

On the other hand, Igarramen et al. presented in 2021 a case report of advanced SCC with an aggressive clinical course that achieved a complete response using chemoradiotherapy as the sole treatment. This successful outcome highlights the potential effectiveness of chemoradiotherapy as a standalone treatment option for advanced maxillary sinus SCC, particularly in scenarios where surgery is not feasible [11].

Radiotherapy is often employed for patients who are not surgical candidates due to tumor extent, comorbidities, or patient preference. Studies have demonstrated that radiotherapy can achieve significant local control and survival rates in selected patients. For instance, a retrospective analysis of 73 paranasal sinus tumors, including 33 SCCs, showed that higher radiation doses correlated with improved local control rates [12]. Similarly, a study of 42 patients with T3-T4N0 maxillary sinus SCC treated with definitive radiotherapy reported five-year overall survival and local control rates of 34% and 29%, respectively, emphasizing the importance of adequate dosing and the potential role of elective neck irradiation in advanced cases [13].

This literature review highlights a wide spectrum of therapeutic options available for this pathology. However, current evidence remains limited in terms of identifying the most appropriate treatment according to disease stage, with few studies directly comparing similarly effective approaches in equivalent clinical scenarios. Our case presents an early-stage SCC (stage II) successfully treated with stereotactic radiosurgery, demonstrating favorable clinical and radiologic outcomes. Nonetheless, it should be emphasized that the current findings are derived from a one-year follow-up, and thus, continuous and careful long-term surveillance is necessary, given the potential risk of recurrence. To the best of our knowledge, this is the first report in the English-language literature documenting a case of T2N0M0 SCC managed with this modality, with both clinical and imaging-confirmed remission after a one-year follow-up period.

## Conclusions

SCC of the maxillary sinus remains a rare and challenging malignancy due to its nonspecific clinical presentation, proximity to critical anatomical structures, and frequent late-stage diagnosis. While surgical resection followed by adjuvant therapy continues to be the mainstay of treatment, patient-specific factors such as tumor stage, comorbidities, and personal preferences may necessitate alternative approaches.

This case highlights the feasibility and potential efficacy of stereotactic radiosurgery using the ZAP-X system as a non-invasive alternative to surgery for early-stage (T2N0M0) maxillary sinus SCC. The patient demonstrated sustained clinical and radiological remission after one year of follow-up, with preservation of function and minimal treatment-related morbidity.

To our knowledge, this is the first reported case in the English-language literature of an early-stage SCC of the maxillary sinus managed exclusively with stereotactic radiosurgery. This case adds to the growing body of evidence supporting individualized, organ-preserving strategies in selected patients, particularly when conventional surgery and/or chemotherapy is declined or contraindicated. Further studies are warranted to explore the long-term outcomes, optimal dosing strategies, and patient selection criteria for stereotactic radiosurgery in sinonasal malignancies.
